# Successful control of *Triatoma dimidiata* with residual application of a microencapsulated formulation of pirimiphos-methyl (Actellic 300CS) in southeast Mexico

**DOI:** 10.1371/journal.pntd.0013311

**Published:** 2025-08-29

**Authors:** Carlos Arisqueta-Chablé, Wilbert Bibiano-Marín, Azael Che-Mendoza, Guillermo Chan-Perez, Henry Ramos-Jiménez, Anuar Medina-Barreiro, Norma Pavía-Ruz, Juan Ortiz-Rivera, Jorge Palacio-Vargas, Fabian Correa-Morales, Hugo Delfín-González, Gabriela González-Olvera, Héctor Gómez-Dantés, María Jesús Sánchez, Gonzalo Vázquez-Prokopec, Pablo Manrique-Saide

**Affiliations:** 1 Unidad Colaborativa para Bioensayos Entomológicos, Campus de Ciencias Biológicas y Agropecuarias-Universidad Autónoma de Yucatán, Mérida, México; 2 Servicios de Salud de Yucatán (SSY), Mérida, México; 3 Centro de Investigaciones Regionales “Dr. Hideyo Noguchi”, Universidad Autónoma de Yucatán, Mérida, México; 4 Centro Nacional de Programas Preventivos y Control de Enfermedades (CENAPRECE) Secretaría de Salud México, Ciudad de México, México; 5 Centro de Investigación en Sistemas de Salud, Instituto Nacional de Salud Pública, Cuernavaca, México; 6 Organización Panamericana de la Salud (OPS), Ciudad de México, México; 7 Department of Environmental Sciences, Emory University, Atlanta, GeorgiaUnited States of America; Fundacao Oswaldo Cruz Instituto Rene Rachou, BRAZIL

## Abstract

**Background:**

*Triatoma dimidiata*, the main vector of *Trypanosoma cruzi* throughout South Mexico and Central America, infest domiciles and peridomestic ecotopes of rural and semi-rural communities. This study reports the effect of the residual application of the organophosphate pirimiphos-methyl in a microencapsulated formulation (Actellic 300CS) for the control of intradomiciliary and peridomestic *T. dimidiata* in the community of Tekik de Regil (hereafter Tekik) in Yucatan, Southeast Mexico.

**Methods:**

From March to October 2022, a two-arm, unblinded entomological trial was performed in Tekik. Timed Manual Collections (TMC) characterized house and peridomicile infestation by *T. dimidiata* prior (baseline) and after the residual spraying (RS) (post-intervention) of a microencapsulated formulation. A total of 120 premises were surveyed (60 positive and 60 negative for *T. dimidiata*), randomly allocated 1:1 to treatment (RS with Actellic 300CS) and control (no RS) arms. Monthly post-spraying entomological surveys (May-October) with TMC were carried out in a random sample of ten houses from each arm. We analyzed the association between the treatment and post-intervention infestations using chi-square contingency tables. The estimated efficacy of the intervention with the 95% Confidence Intervals (CI) was calculated with the efficacy formula, using the Odds Ratio (OD) calculated from a binomial Generalized Linear Mixed Model (GLMM) from the positive premises in the baseline survey and post-intervention, using time as a random effect.

**Results:**

Domestic infestations post-intervention were only detected in the control group (2/60 houses, 3.3%). Cumulative peridomestic infestation was significantly higher in the control arm (31.7%; 19/60) compared to the treatment arm (11.7%; 7/60) (*X*^2^ = 0.007, p < 0.01). The cumulative 6-month estimated efficacy of the intervention (% reduction in treatment versus control arm) was 65% (95% CI: 14%-79%).

**Conclusions:**

A single application of Actellic 300CS reduced *T. dimidiata* infestations by more than 60% for up to 6 months and provides evidence of an alternative formulation suitable for triatomine control in Mexico.

## Author summary

Chemical control of Chagas disease has been focused on the use of pyrethroid formulations for over 40 years. New insecticides and formulations are available but have not been sufficiently evaluated for Triatomine control. This study reports the efficacy of the residual application of a new microencapsulated formulation for the control of *T. dimidiata* in the rural community of Tekik in Yucatan, Southeast Mexico. We surveyed 120 residences and randomly allocated them in a 1:1 in treatment (residual spray) and control (no residual spray) groups. Then, we carried out one application of pirimiphos-methyl in a microencapsulated formulation using manual compression sprayers at a dose of 1g/m^2^. We measured triatomine prevalence and abundance monthly from a random sample of ten houses from each group. Post-intervention entomological surveillance showed that domestic infestations by *T. dimidiata* were only detected in the control group and that the peridomestic infestations were significantly reduced in the treatment group with an estimated efficacy of 65% for 6 months post-intervention. This study provides evidence that Actellic 300CS can reduce *T. dimidiata* infestations and is an alternative for the control of triatomines in Mexico.

## Introduction

Chagas disease or American trypanosomiasis is a neglected tropical disease caused by the protozoan parasite *Trypanosoma cruzi* [1,2]. The main mechanism of transmission of *T. cruzi* is through an insect vector and occurs when humans have contact with the feces of infected hematophagous hemipterans (Family Reduviidae, subfamily Triatominae) [3]. It is estimated that more than 6 million people are infected with *T. cruzi* in endemic areas of 21 countries of the Americas [4]. In Mexico, Chagas disease is considered the most important parasitic disease based on its prevalence and burden [5,6], with estimations as high as 4.1 million people infected [6,7].

*Triatoma dimidiata* (Latreille, 1811) is the main vector of *T. cruzi* and Chagas disease throughout southern Mexico and Central America. *T. dimidiata* can infest and colonize houses and peridomestic structures (used as animal refuges) of urban, suburban, and rural communities [8–11]. Recently, the Mexican Ministry of Health (MMoH), together with the Pan American Health Organization (PAHO), launched an initiative for the implementation of comprehensive and intensified actions to advance the elimination of intradomiciliary vectorial transmission of Chagas disease [12,13]. Vector control with the application of residual insecticides, both intra- and peridomestic, is a key component of the plan to eliminate indoor infestation by *Triatoma* species (including *T. dimidiata*) throughout a stratified list of localities of endemic states [13–17].

In Mexico, the national program for the control and elimination of Chagas disease has traditionally recommended the use of RS with pyrethroid insecticides for the control of triatomine vectors [18]. This recommendation is based on the successful experiences of triatomine control with pyrethroid insecticides in Latin America [14] and the recommendations of the World Health Organization [15] and the Pan American Health Organization [16]. Pyrethroids (e.g., deltamethrin, lambda-cyhalothrin, cyfluthrin, and cypermethrin) became the main insecticides used operationally for control of Triatominae since the mid 1980’s, because of their efficacy and persistence at low doses, as well as the low environmental risk associated with their use [14,15,18,19]. However, while efficacious indoors, pyrethroids show poor residuality and efficacy in peridomestic habitats [20–22].

Thanks to innovations for malaria control, new chemical groups and formulation technologies are now available for public health use, e.g., organophosphates, neonicotinoids, and pyrroles [23–27]. Some of these new insecticide/formulation alternatives are available and already recommended for residual spraying and vector control in Mexico [24,28,29], but they have not been sufficiently evaluated for *T. dimidiata* [30]. Efficacy of vector control as part of this Mexican initiative for the elimination of intradomiciliary vectorial transmission of Chagas disease can be successfully achieved with insecticide formulations with long-lasting residual efficacy to which triatomine vectors are susceptible. More broadly, the lack of updated WHO guidelines about the potential of such novel residual insecticide formulations for Triatomine control emerges from a lack of evidence from entomological field trials evaluating their efficacy under field conditions. Here, we report the results of a field randomized trial quantifying the efficacy of a microencapsulated formulation of the organophosphate insecticide Actellic 300CS on *T. dimidiata* infestation in rural dwellings in a community in southern Mexico.

## Materials and methods

### Ethics statement

This study was part of the integrated activities conducted by the Vector Control Program for Chagas Disease under the Ministry of Health [31]; therefore, an Institutional Review Board evaluation was not required.

### Study site

**Fig 1:**
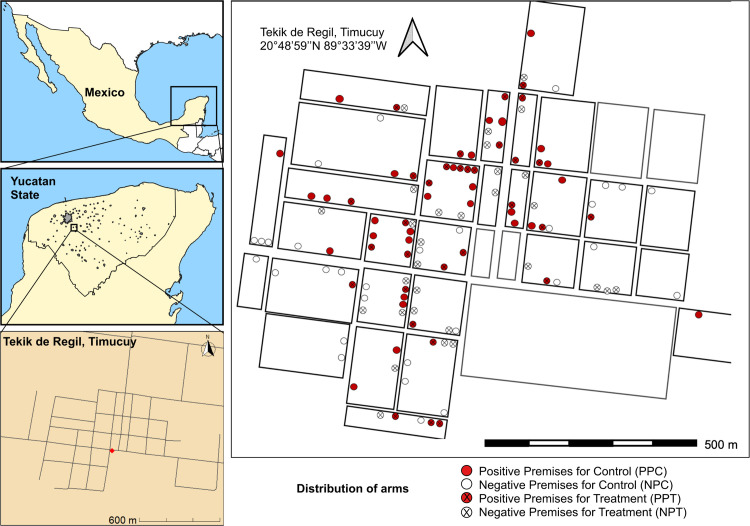
Location of the study site in Tekik de Regil, Yucatán, México, and distribution (sketch) of the study arms: Control (PPC and NPC), red spots are premises positive, and the white spots are premises negative for *Triatoma dimidiata* infestations. The spots with crosses receive treatment (PPT and NPT). The base map was created in QGIS 3.36.1 Maidenhead (qgis.org), using layers OpenStreetMap (http://www.openstreetmap.org/), under the Open Database License (https://www.openstreetmap.org/copyright).

The study included premises located in the locality of Tekik (20°48′59″N 89°33′39″W), Municipality of Timucuy, on the outskirts of the city of Merida in the Yucatán Peninsula (southeastern Mexico) (Fig 1). Tekik was selected in consensus with the local Ministry of Health due to the historical presence of the triatomine vector and the human transmission of *T. cruzi* in the area [11,32,33] and willingness of the inhabitants of the community to participate in the study.

Tekik is a semi-rural locality with 2,080 inhabitants who live in 484 homes [34]. Located at an altitude of 10 m above sea level, it has a warm climate with an average annual temperature of 26°–28°C (36°C max–18°C min), a relative humidity of 70–75%, and a rainy season (May/June to October) with a rainfall of 882.5 mm, and a dry season (November/December to April) with a rainfall of 167.9 mm [35]. Tekik has characteristics like those found in other suburban/rural communities in Yucatán [11,36]. Houses are typically one-story, made of cement blocks (with or without plastered and painted walls), and most have cement floors and ceilings. Generally, houses have an outdoor kitchen with a metal sheet roof where the owners cook with firewood and a laundry area surrounded by a large peridomicile/backyard (patio, on average 200 m^2^) generally delimited by a fence of stacked rocks (1.25 m high) and with arboreal vegetation where domestic animals live (mainly dogs and chickens). Animal pens and chicken coops are the most common peridomestic structures in the area [37].

### Baseline entomological surveys

**Fig 2:**
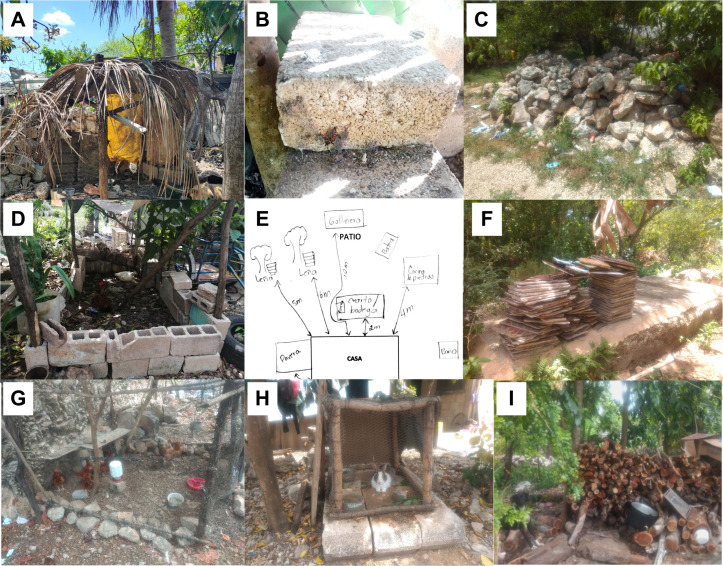
Examples of the main peridomestic ecotopes where *T. dimidiata* was found and collected at the study site. **A)** Doghouse, **B)** Concrete blocks, **C)** Piled rocks **D)** Chicken coop made of concrete blocks, **E)** Example of a sketch of premises with their peridomestic ecotopes, **F)** Piled construction material, G) traditional chicken coop made with wire mesh, rocks, and wooden pillars, **H)** Rabbit hutch using concrete blocks, I) piled firewood in the backyard. Photo by Arisqueta-Chablé.

To determine the level of infestation of *T. dimidiata* in Tekik, we conducted a baseline entomological survey (BL), performed by five teams of two skilled entomologists from the Collaborative Unit for Entomological Bioassays (UCBE-UADY) and the local Ministry of Health, Servicios de Salud de Yucatán (SSY). From March 5^th^ to 19^th^, 2022, written informed consent was obtained from one adult in each household. Households, where the owners declined participation, were noted and excluded from intervention activities, including spraying and post-intervention entomological surveys. The prevalence (presence of at least one triatomine bug) and number (abundance) of *T. dimidiata* were recorded by TMC [13,26]. We define “premises” as the whole intradomiciliary and the peridomestic area. The intradomiciliary collections were performed using a flashlight to search inside cracks and crevices throughout the structure of the buildings, inside closets, behind pictures on walls, and especially under furniture and bedding for 30 minutes. Peridomestic collections lasted 30 min per premises and involved ecotopes known to be habitats of *T. dimidiata* such as chicken coops, dog houses, stone fences, and firewood piles [13,33,36,38]. As described in the manual for field testing and evaluation of insecticides against domestic vectors of Chagas disease, TMC catches were supplemented with community participation by providing resealable bags weekly and asking them to collect triatomines (termed householder collections, HC) [39]. The status of premises (intradomiciliary or peridomestic) for *T. dimidiata* was recorded (positive or negative). A sketch of the house and the surrounding peridomicile (including a census of ecotopes/potential ecotopes) was made for each premises (Fig 2).

### Pre-treatment characterization of the insecticide susceptibility profile in the local population of T. dimidiata

Specimens of *T. dimidiata* collected during the BL were transported alive to the UCBE-UADY insectary. Parental groups with adult specimens were used to obtain eggs and nymphs I (F1). We performed topical application bioassays with diagnostic doses (DD) calculated from the LD_99_ (lethal doses) of a laboratory strain of *T. dimidiata* to determine the susceptibility profile of the triatomine population from Tekik [40]. The DD of four insecticide active ingredients: Deltamethrin (3.8 ng/insect), Alpha cypermethrin (60.6 ng/insect), Bendiocarb (107.5 ng/insect), and Pirimiphos-methyl (223.3 ng/insect), the insecticide to be tested in this field study. A micro-drop (0.2 µl) of the active ingredient of each insecticide diluted in acetone was deposited in the dorsal area of the abdomen of three groups of 10 nymphs I (unfed, three days old, and weighing 0.9-1.2 milligrams) with a 10 µl Hamilton micro-syringe with a repetitive dispenser (PB600–1). Insects were afterwards placed in clean, wide-mouth jars with folded paper and mesh. Mortality data were recorded after 72 hours. Insects that could not walk on a circular paper (9 cm) were considered dead [40].

### Study design

After assessing the infestation levels of *T. dimidiata* in Tekik at BL, we conducted a two-arm (RS versus untreated control), unblinded entomological trial. A total of 120 premises (60 positive for *T. dimidiata*, and 60 negative) were included for stratified random allocation of treatment and control arms 1:1 for premises found infested by *T. dimidiata* and for negative premises (Fig 1). Such allocation led to the generation of four study groups for analysis (Fig 1), as follows: the control arm consisted of a group of 30 Positive Premises for Control (PPC) and 30 Negative Premises for Control (NPC), and the treatment arm: 30 Positive Premises with Treatment (PPT), and 30 Negative Premises with Treatment (PNT) (Fig 1). The allocation of premises to each arm was done using a random number generator. The primary endpoint for analysis was to quantify the entomological reduction in premises infested after the RS of the long-lasting microencapsulated formulation (Actellic 300CS, Syngenta, Basel, Switzerland) compared to the untreated control arm and the prevention of new infestations in negative premises. Given that the search for *T. dimidiata* often leads to the destruction of the habitats where they are found and the extraction of all bugs infesting them (e.g., piles of rock or small chicken coops), we modified our approach for entomological follow-up surveys to capture both the monthly and cumulative impact of the intervention. Instead of sampling all the premises longitudinally (which would have led to the removal of many populations on the first post-straying entomological survey), we conducted monthly collections using only the TMC method in a random subset of premises to achieve one sampling per house by the end of the study (6 months). This led to dividing the total of 120 premises into monthly entomological collections of 10 premises per month, per study arm. We assumed a high probability of premises being positive at baseline and remaining positive for 6 months; as such, balancing our trial to quantify efficacy on positive and negative premises at baseline allowed estimating the impact of the intervention on the rates of re-infestation and potential new infestations, respectively.

### Residual spraying

**Fig 3:**
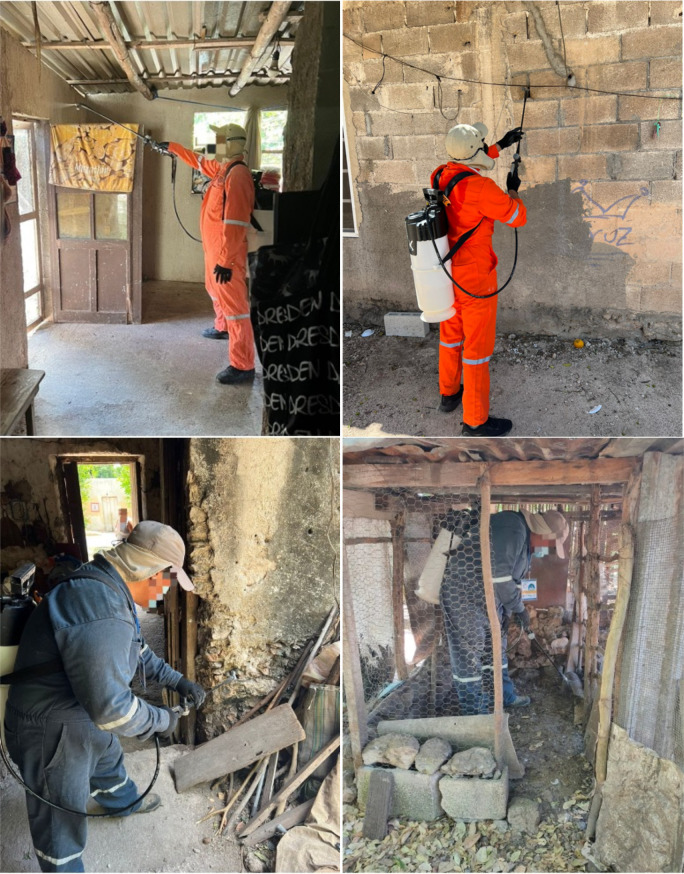
Residual spraying applied inside and outside the houses, in positive/potential ecotopes of triatomines. Photo by Arisqueta-Chablé.

After the characterization of the insecticide susceptibility profile of local *T. dimidiata*, we carried out the RS of Actellic 300CS (pirimiphos-methyl 28.16%, 833-ml bottle, micro-encapsulated suspension, Syngenta of Mexico), within houses and all possible refuges of triatomines in peridomicile using an IK-Vector Control Super manual compression sprayer with 8002EVP nozzle and Goizper low-pressure flow control valve (1.5 bar outlet pressure) (Fig 3). From April 19^th^ to 21^st^ 2022, five teams composed of personnel from SSY and entomologists from the UCBE-UADY. The staff wore uniforms and appropriate personal protective equipment, had been previously trained, and had experience in the application of the IRS protocols used for malaria adapted to TIRS for the urban control of *Ae. aegypti* [41–43]. In the interior of the houses, we carried out the RS application of insecticide at a dose of 1g/m^2^; the technical parameters for the spray (distance of the rod to the wall, speed, pressure, etc.) were kept as they are used in the classic indoor residual spraying (IRS) (40). The dose used for the peridomestic area was 1 g/m^2^ for flat surfaces (walls, wooden panels, cardboard sheets), and for irregular surfaces such as stones, holes, cracks, the interior of concrete blocks, and small spaces, an estimated dose of 1–2 g/m^2^ was applied considering the difficulty of maintaining a constant distance and speed of spraying on them.

For the indoor RS, the classic method of vertical strips was used to spray all internal surfaces with reinforcement in cracks and fissures in the wall and peridomestic area [21,39]. Houses were sprayed on the internal and external wall surfaces, around door and window openings, eaves, and roof edges. In addition, RS was applied to all peridomestic ecotopes identified during the BL study as potential ecotopes and the perimeter wall of animal pens. (1m high). Some modifications were made to increase acceptability by homeowners: personal belongings were not taken out of the home and furniture was not completely moved away from the walls, therefore only exposed walls were sprayed; picture frames and other belongings hung on the walls were kept unless the owner decided to remove them; kitchens were not sprayed to minimize the risk of food contamination; when possible, domestic animals were removed just before spraying, but insecticide was never applied to them, nor applied to feeders or animal drinkers. To minimize exposure risks, residents were asked to stay out of the home for 30 minutes after the RS. All treated premises were revisited to ask whether residents observed any negative effects indoors and outdoors. Additionally, six months after the intervention, all positive premises were offered RS to address any remaining *T. dimidiata* infestations.

### Community acceptance

A semi-structured interview was conducted with 60 participating heads of family from the community (October to December 2022). The interview included quantitative and qualitative questions that addressed several topics, such as the profile of participants, reasons for participating in the project activities, characteristics of triatomine, and the perception of the benefits and disadvantages of RS.

### Data analysis

We analyzed the post-intervention infestations by *T. dimidiata* throughout six months (May-October) collected by TMC. The infestation rate was calculated by dividing the number of infested sites by the total number of surveyed sites and multiplying by 100 at the different levels (study arms, intradomiciliary, peridomestic, and ecotopes). We performed a chi-square contingency analysis to examine the impact of RS on infestations. Finally, we assigned the status of the surveyed premises as a binary variable (1 = positive, 0 = negative). To calculate the efficacy of the entire intervention, we performed a binomial GLMM using survey time (from 1 to 6 months) as a random effect to quantify the significance of the difference between arms (fixed effect), then we calculated the odds of detecting a positive premise in the treatment arm compared to the control. The efficacy of the intervention was calculated using the OR with the 95% CI) was, derived from the fixed effects of the GLMM, and applied in the adapted intervention efficacy formula: Efficacy (%) = (1- OR) *100. All analyses were conducted using the software R version 4.2.2.

## Results

### Baseline characteristics from entomological survey

During the BL entomological survey, out of a total of 471 premises visited 226 (48%) were accessible (the remaining locations were closed, refused or were vacant/non-residential, e.g., shops, schools, churches etc.). *T. dimidiata* was detected in 27% (61/226) of the premises visited. The intradomiciliary infestation represented 6.6% (4/61) (each house with one triatomine bug), and 93.4% (57/61) were infested in the peridomicile. Most of the positive premises were infested by immature stages of *T. dimidiata* (colonized): 29.5% (18/61) of those premises were found positive for adults, 21.3% (13/61) for nymphs, and 49.1% (30/61) for both stages. The TMC method was the most sensitive to detecting positive premises (93.4%) and collected the most Triatomine bugs with 96.7% (Table 1). In total, 330 specimens of *T. dimidiata* were collected: 62.7% were nymphs, and 37.3% were adults (Table 2). Most of the specimens (98.8%) were collected in the peridomicile. The most abundant immature stage collected was Nymph V with 19.3%, followed by the adults: males 18.8% and females 18.4%. The average number of triatomines collected by TMC was 5.6 (319/57), with 1.9 adults/per positive house and 3.6 nymphs/per positive house (Table 1). The average number of triatomines collected by HC per positive house was 2.8 (11/4), but this method was only able to detect adults (Table 1).

**Table 1:** Summary of premises infested by *Triatoma dimidiata* collections by method (TMC = Timed Manual Collections, HC = Householder Collections) and site at baseline survey (pre-intervention- March 2022). Infestation refers to premises positive to triatomine.

Collection method	Infestation		Intradomiciliary		Peridomestic		Total (%)
	Intradomiciliary (%)	Peridomestic (%)	Adults	Nymphs	Adults	Nymphs	
TMC	2 (3.5)	55 (96.5)	1	1	111	206	**319 (96.7)**
HC	2 (50)	2 (50)	2	0	9	0	**11 (3.3)**
**Total**	**4 (6.6)**	**57 (93.4)**	**3**	**1**	**120**	**206**	**330 (100)**

**Table 2:** Summary of *Triatoma dimidiata* abundance by life stage, sex, and site collections at baseline survey (pre-intervention- March 2022).

Site	Adults collected		Total adults (%)	Total Nymphs (%)					Total Nymphs (%)	Total by site (%)
	♂	♀		N I	N II	N III	N IV	N V		
Intradomiciliary	1	2	**3 (2.5)**	0	0	0	0	1	**1 (0.5)**	**4 (1.2)**
Peridomestic	61	59	**120 (97.5)**	34	33	39	37	63	**206 (99.5)**	**326 (98.8)**
**Total**	**62**	**61**	**123 (100)**	**34**	**33**	**39**	**37**	**64**	**207 (100)**	**330 (100)**

A total of 598 peridomestic ecotopes were counted and sampled, of which 12.9% (77/598) were positive for *T. dimidiata*. Chicken coops were the most productive and frequently infested structure with 240 triatomine bugs (76.2% of the total specimens collected) and 20.7% (54/261) of positivity (Table 3). Dog houses and woodpiles were important refuges of *T. dimidiata* as well (Table 3). Susceptibility bioassays were conducted on ~100 individuals for each insecticide tested. A control group of nymphs was also kept and observed. All individuals exposed to diagnostic doses of the four insecticidal active ingredients (alpha-cypermethrin, deltamethrin, bendiocarb, and pirimiphos-methyl) were classified as susceptible (S1 Table).

**Table 3:** Summary of peridomestic ecotopes infested by *Triatoma dimidiata* at baseline survey (pre-intervention, March 2022).

Ecotopes	Positive (surveyed)	Infestation %	Triatomine (%)
Chicken coop	54 (261)	20.7	240 (76.2)
Dogs house	7 (202)	3.5	28 (8.9)
Woodpile	6 (51)	11.8	4 (1.3)
Pile of rock	3 (41)	7.3	9 (2.9)
Piled material	4 (24)	16.7	16 (5)
Rabbit hutch	3 (19)	15.8	18 (5.7)
Total	**77 (598)**	**12.9**	**315 (100)**

### The overall impact of residual spraying on T. dimidiata infestations

A total of 129 specimens of *T. dimidiata* were sampled post-spraying in 28 premises infested out of the 120 surveyed. The population structure was 80.6% (104/129) of the specimens were immature, and 19.4% (25/129) were adults. The premise infestation by arms revealed that the control arm was significantly the most infested, with 35% (21/60), three times the infestation rate compared to the treatment arm, with 11.7% (7/60) (*X*^*2*^ = 9.13; *P* = 0.0025). The premises infestation by study group was recorded as follows: in the control arm, the PPC group recorded 66.7% (20/30) of premises infested, and the NPC group recorded 3.3% (1/30) of positive premises. In the treatment arm, the PPT group recorded 23.3% (7/30) positive premises; in the NPT group, no positive premises were found out of 30 surveyed (S2 Table).

### *Impact of residual spraying on intradomiciliary infestations of T. dimidiata surveyed* by TMC

According to the site-infested premises, we found that 1.7% (2/120) of all the premises studied were positive for *T. dimidiata* indoors. The proportion of positive premises intradomiciliary was 7.1% (2/28). The infestation rate by arm represents 3.3% (2/60) for the control against the treatment arm, where no intradomiciliary infestations were found (S2 Table). This low number of positive domiciles prevented any statistical analysis.

### *Impact of residual spraying on peridomestic infestations of T. dimidiata surveyed* by TMC

**Fig 4:**
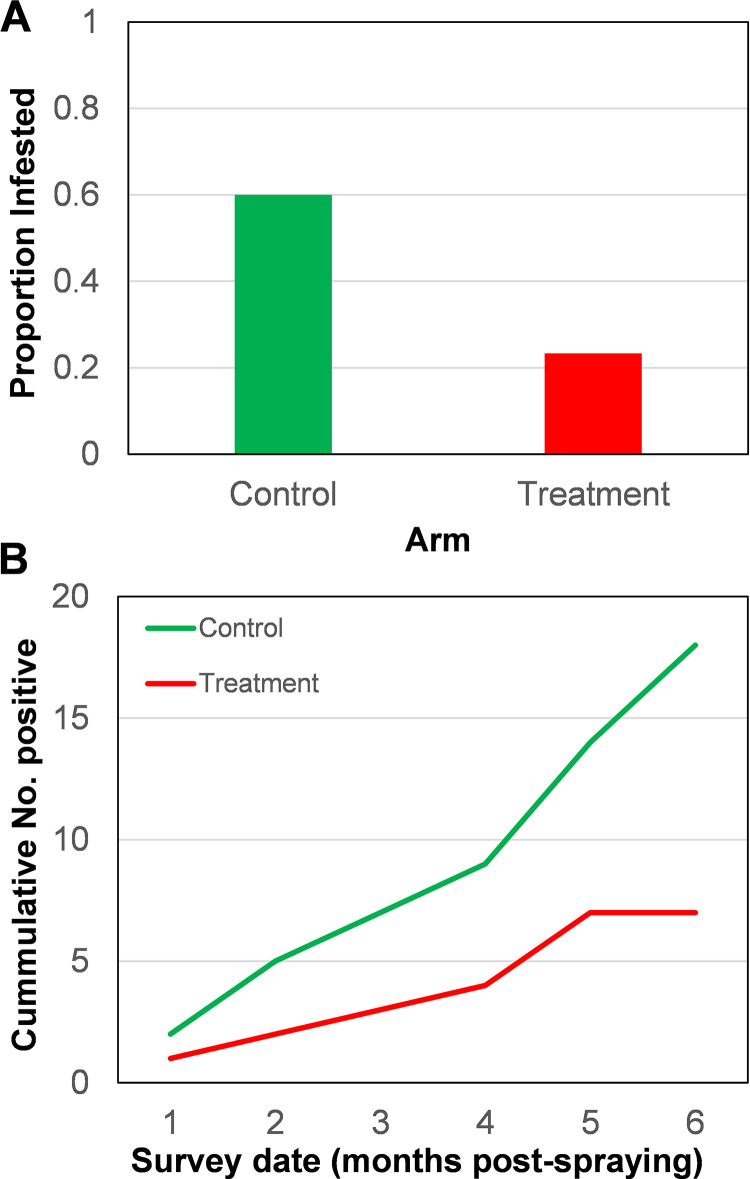
(A) Proportion of peridomiciles detected infested by *Triatoma dimidiata* at baseline found positive during the 6-month follow-up post-spraying. (B) Cumulative number of peridomiciles was found positive by *T. dimidiata.*

Throughout six months post-intervention (May- October 2022), 26 of the 120 premises surveyed were infested in the peridomicile. Also, the proportion of positive peridomiciles represented 92.8% (26/28). The cumulative peridomestic infestation by arm was significantly lower at 11.7% (7/60) in the treatment arm compared to the control (31.7%; 19/60) (X^2^ = 7.07, *P* = 0.0078) (See S2 Table). The proportion of positive peridomiciles at BL found infested in the control arm post-intervention was significantly higher, 60% (18/30), than the treatment arm, 23.3% (7/30) (Fig 4A; X^2^ = 10.4, P = 0.0013). Interestingly, the impact of the intervention occurred throughout the 6 months post-spraying, was observed in the lower cumulative number of peridomiciles detected positive over time in the treatment arm (Fig 4B).

From all the triatomine collected, 98.4% of triatomine bugs (127/129) were found in the peridomicile, while only 1.6% (2/129) were collected in the intradomicile, primarily from premises infested at BL. At the start of the study, negative premises were included in both the control and treatment arms. Notably, only the study group NPC of the control arm transitioned from negative to positive after the intervention, whereas the study group NPT remained consistently negative (S2 Table).

The majority of bugs were collected from the control arm (92.2%, 119/129), while 7.8% (10/129) was found in the treatment arm (S3 Table). Within the control arm, the population structure was 82.3% (98/119) immature stages and 17.6% (21/119) adults, and the treatment arm presented 60% (6/10) immature stages and 40% (4/10) adults (S3 Table). The sex ratio of the adults collected in both arms was 64% (16/25) males and 36% (9/25) females. Within the control arm, the sex ratio was 66.6% (14/21) males, while 33.3% (7/21) were females. In the treatment arm, 50% (2/4) were males and 50% (2/4) were females (S3 Table). Of all the nymphs collected, 94.3% (98/104) were in the control arm, and 5.7% (6/104) were in the treatment group. In the control group, the most abundant immature stage was nymph V with 42.9% (44/98), followed by nymphs IV with 28.6% (30/98) and III with 15.4% (16/98) (S3 Table).

We detected 26 ecotopes infested from 373 peridomestic ecotopes surveyed during the post-intervention phase, a detailed description of the ecotopes infested is provided (see Table 4). Four types of ecotopes were positive: goat/sheep pens, the pile of rocks, dog houses, and chicken coops, which again were the most frequently infested ecotopes, consistent with the findings from the BL survey. Overall, it can be observed that both infestations and the number of triatomines collected in the treatment arm were lower compared to the control arm. The proportion of positive ecotopes per arm in the treatment group was 27% (7/26), compared to the control arm, 73% (19/26).

**Table 4:** Infestations of peridomestic ecotopes by *Triatoma dimidiata* post-intervention (May-October 2022) in the control and treatment arms. The type and number of positive ecotopes (surveyed), percentage of infestation, percentage of triatomine bugs and *P-value* of the Chi-square contingency table analysis are listed. signif. codes: *P < 0.05, **P < 0.10.

Ecotopes	Control			Treatment			*P-value*
	Positive (Surveyed)	Infestation (%)	Triatomine (%)	Positive (Surveyed)	Infestation (%)	Triatomine (%)	
Chicken coop	12 (84)	14.3	104 (88.4)	6 (101)	5.9	9 (90)	0.09737**
Dogs house	5 (43)	11.6	9 (8)	1(40)	2.5	1 (10)	0.1086
Pile of rocks	1 (13)	7.7	1 (0.8)	0 (8)			
Goat/sheep pen	1 (3)	33.3	3 (2.6)	0 (1)			
Rabbit hutch	0 (3)			0 (5)			
Piled material	0 (8)			0 (10)			
Pile firewood	0 (17)			0 (12)			
Pigpen	0 (8)			0 (9)			
Storeroom	0 (4)			0 (2)			
Latrine	0			0 (2)			
Total	**19 (183)**	**10.4**	**117**	**7 (190)**	**3.7**	**10**	0.01948*

The result of the GLMM quantifying the impact of the intervention on *T. dimidiata* infestation is shown in Table 5. A significant reduction in infestation, indicative of a strong intervention effect, was quantified. Furthermore, the estimated efficacy of the residual insecticide application on reducing *T. dimidiata* infestation was 65% (95% CI = 14%-79%).

**Table 5:** Random-effects logistic regression model (GLMM) of *Triatoma dimidiata* infestation odds 6 months after Actellic 300CS residual spraying. S.E. standard error, signif. codes: * P < 0.05, **P < 0.10.

Random effects				
Groups name	Variance	S. E.		
Time (Intercept)	2.081e-10	0.00001443		
Fixed effects	**Estimate**	**S. E.**	**Z value**	**Pr (>|z|)**
(Intercept)	-0.4055	0.2216	-1.830	0.0673**
Treatment	-1.0498	0.4331	-2.424	0.0154 *

### Community acceptance

During the post-intervention interviews, residents of all treated premises demonstrated a favorable disposition toward the use of insecticides and expressed acceptance of the methodologies implemented during the visits. As effects of indoor application, 51.7% (31/60) reported dead insects and spiders (but not triatomines), 25% (15/60) fewer mosquitoes in homes and surroundings, and no insecticide intoxications were reported in people or animals. Of all the participating treated premises, 98.3% (59/60) would be willing to receive spraying inside their homes again, and 100% of the homes would be willing to receive perimeter and outside spraying of their homes. Only two premises (2/60, 3.3%) reported that the insecticide had a strong odor; however, they indicated a willingness to apply it outdoors again.

## Discussion

We report the entomological impact of the residual application of the organophosphate pirimiphos-methyl, in a microencapsulated formulation (Actellic 300CS) on *T. dimidiata* infestations in Tekik, Yucatán, emphasizing the treatment of identified ecotopes in the BL phase and surveyed throughout six months of post-intervention. Peridomestic infestations accounted for more than 90% of all infestations observed throughout the study. During this period, we identified a low level of vector domiciliation compared to peridomestic infestations, consistent with findings reported in other studies [44,45]. In general, domestic infestations detected by TMC were related to the presence of chickens and firewood inside the houses, risk factors that have been pointed out by other authors [38,44,46–49]. Some authors mention that peridomestic infestations represent a significant risk for negative homes in the surrounding area and for homes with peridomestic infestations to acquire intradomiciliary infestations over time [21]. Other studies have found a significant relationship between the presence of animal pens, especially dogs and chickens (which were important in this work), on peridomestic infestations by *T. dimidiata* and other triatomines [11,20,36,47,50–52], and the risk that these man-made ecotopes represent on triatomine infestations in the peridomicile [20,53–55]. Due to its presence in the natural environment surrounding houses, *T. dimidiata* can re-infest/colonize treated structures shortly after an intervention [56–59].

Given the low chance of detecting *T. dimidiata*, we focused our analyses on the subset of premises that were infested at BL (60) to quantify the efficacy of the intervention. When only premises found positive by *T. dimidiata* at BL are analyzed (excluding the NPC and NPT study groups), the cumulative number of positive units increased significantly more in the control compared to the treatment arm, indicating a strong intervention effect on *T. dimidiata*. The intervention demonstrated an estimated efficacy of 65% (95% CI: 14–79%) for up to 6 months, a notably high value given the fact that most of infestations were peridomestic. However, the relatively wide 95% CI suggests a potential limitation which may be attributable to the limited number of infested premises at baseline. The results of the residual effect in this work were consistent with other microencapsulated studies with triatomines [60–65]. Although RS with Actellic 300CS was applied thoroughly, at the end of the study, one-third of re-infested/colonized peridomestic structures were found in the treatment group, similar to previously documented RS interventions for *T. dimidiata* and other vectors of Chagas disease [20,56,58,66,67]. Since the local population of *T. dimidiata* was tested as susceptible to pirimiphos-methyl, some hypotheses that can explain the few re-infestations/colonization events are: the vector avoiding insecticide exposure or egg hatching after residual effect of the insecticide waned; operating failures including sub-optimal insecticide applications or presence of triatomine bugs in difficult to treat surfaces such as tree trunks, plastered stone walls and stone fences [56,66,68–71]. In addition, these bulky structures are commonly used by people to establish chicken coops, dog houses, and other typical animal pen structures in the region, which were important in this work as residual foci structures.

In this work, we implemented TMCs for entomological surveillance in both arms each month instead of sampling all premises monthly. Since *T. dimidiata* is a triatomine species with a long-life cycle and low densities, the impact of the removal effect on *T. dimidiata* populations could be large, leading to biases in our estimates of intervention residual efficacy [16,45,68,72]. Also, TMCs allowed us to observe the insecticidal effect applied directly to the ecotopes identified as positive rather than mixing with the HC method since intrusive adult triatomines are common in Yucatan and these infestations can be misinterpreted as chemical control failure [10,57]. In addition, the proportion between the adults and immature stages collected was different from that reported in other studies in Yucatan [9,11,32,73]. The difference between these proportions of stages is mainly due to the collection methods. Householder collections are useful for detecting domestic infestations of some *T. dimidiata* adults per house, but they are not able to detect densely infested peridomestic ecotopes. Population demographic parameters indicate that *T. dimidiata* reproduces 2.7 to 30 times more in the peridomicile than indoors [38]. Peridomiciles are, thus, a key epidemiological environment not only because of the large number of bugs in them but also because of the presence of *T. cruzi* reservoirs such as opossums, dogs, and small rodents that can maintain the parasite in an area even at low density of bugs [11,55,74]. Therefore, focusing vector control on peridomestic habitats by using long-lasting insecticide formulations may lead to sustainable interruption of parasite transmission by eliminating infected *T. dimidiata* bugs that later disperse indoors and have contact with people [21,22,75].

Chemical control through residual spraying is the most cost-effective and long-lasting intervention against triatomine infestations [67,76,77], and together with blood screening for transfusion has led to an estimated reduction in Chagas disease incidence of 70% in the Southern Cone countries and 67% in the American continent [78,79]. In this context, interventions with pyrethroids against *T. dimidiata* have shown different results in efficacy and residuality, although generally, after 3 or 6 months, reapplication is needed [56–58,80–83], like the conventional non-pyrethroid insecticide formulations [30,80]. However, microencapsulated insecticide formulations such as Actellic 300CS offer a long-lasting standardized application method (important for vector control programs) while maintaining similar efficacy and residual effect to insecticidal paints, with fewer applications than conventional insecticide formulations [65].

This study demonstrates that high-quality, non-pyrethroid microencapsulated formulations offer significant public health benefits by effectively controlling peridomestic *T. dimidiata* populations for up to six months. Achieving a 65% efficacy rate in an endemic area with a single application represents a substantial impact, especially considering the persistent presence of natural populations of *T. dimidiata* that act as continuous reinfestation sources. Integrating RS into a comprehensive vector management plan with alternative control strategies could provide sustainable control of *T. dimidiata* populations and contribute to the interruption of *T. cruzi* transmission.

## Conclusions

We provide evidence of the entomological impact of a single application of a microencapsulated insecticide formulation that can reduce *T. dimidiata* peridomestic infestations by more than 60% for up to 6 months as an alternative non-pyrethroid formulation suitable for triatomine control in Mexico.

## Supporting information

S1 Table Pre-treatment characterization of the insecticide susceptibility profile in nymph I of *Triatoma dimidiata* (Tekik strain). DD = Diagnostic Dose; ng/i = nanograms per insect; n = number of triatomines tested.
(DOCX)

S2 Table Summary of premises positive and abundance of *Triatoma dimidiata* by site and study groups. PPC = positive premises for control, NPC = negative premises for control, PPT = positive premises for treatment, NPT = negative premises for treatment.
(DOCX)

S3 Table Summary of *Triatoma dimidiata* population structure post-intervention (May-October 2022) by arm. Males = ♂, Females = ♀.
(DOCX)
