# Supplementary material for: Successful control of Triatoma dimidiata with residual application of a microencapsulated formulation of pirimiphos-methyl (Actellic 300CS) in southeast Mexico
Source: PLoS Negl Trop Dis. 2025 Aug 29;19(8):e0013311. doi: 10.1371/journal.pntd.0013311 (PMC12416830; doi:10.1371/journal.pntd.0013311)
Supplement: S1 Table — DD = Diagnostic Dose; ng/i = nanograms per insect; n = number of triatomines tested. (DOCX) [file pntd.0013311.s001.docx]

S1 Table. Pre-treatment characterization of the insecticide susceptibility profile in nymph I of *Triatoma dimidiata* (Tekik strain). DD = Diagnostic Dose; ng/i = nanograms per insect; n = number of triatomines tested.

| Insecticide | Chemical group | DD  ng/insect | n | Mortality  % | Status |
| --- | --- | --- | --- | --- | --- |
| Alfa cypermethrin | Pyrethroid | 60.6 ng/i | 99 | 100 | Susceptible |
| Deltamethrin | Pyrethroid | 3.79 ng/i | 102 | 100 | Susceptible |
| Bendiocarb | Carbamate | 107.5 ng/i | 108 | 100 | Susceptible |
| Pirimiphos methyl | Organophosphate | 223.3 ng/i | 102 | 100 | Susceptible |
