# Supplementary material for: Successful control of Triatoma dimidiata with residual application of a microencapsulated formulation of pirimiphos-methyl (Actellic 300CS) in southeast Mexico
Source: PLoS Negl Trop Dis. 2025 Aug 29;19(8):e0013311. doi: 10.1371/journal.pntd.0013311 (PMC12416830; doi:10.1371/journal.pntd.0013311)
Supplement: S2 Table — PPC = positive premises for control, NPC = negative premises for control, PPT = positive premises for treatment, NPT = negative premises for treatment. (DOCX) [file pntd.0013311.s002.docx]

S2 Table. Summary of premises positive and abundance of *Triatoma dimidiata* by site and study groups. PPC=positive premises for control, NPC=negative premises for control, PPT=positive premises for treatment, NPT=negative premises for treatment.

| SITE | CONTROL | | | | TREATMENT | | | |
| --- | --- | --- | --- | --- | --- | --- | --- | --- |
|  | **PPC** | | **NPC** | | **PPT** | | **NPT** | |
|  | **Infestation** | **Triatomine bugs** | **Infestation** | **Triatomine bugs** | **Infestation** | **Triatomine bugs** | **Infestation** | **Triatomine bugs** |
| Intradomiciliary | 2 | 2 | 0 | 0 | 0 | 0 | 0 | 0 |
| Peridomestic | 18 | 113 | 1 | 4 | 7 | 10 | 0 | 0 |
| Total (surveyed) | **20 (30)** | **115** | **1(30)** | **4** | **7 (30)** | **10** | **0 (30)** | **0** |
