# Supplementary material for: Successful control of Triatoma dimidiata with residual application of a microencapsulated formulation of pirimiphos-methyl (Actellic 300CS) in southeast Mexico
Source: PLoS Negl Trop Dis. 2025 Aug 29;19(8):e0013311. doi: 10.1371/journal.pntd.0013311 (PMC12416830; doi:10.1371/journal.pntd.0013311)
Supplement: S3 Table — Males = ♂, Females = ♀. (DOCX) [file pntd.0013311.s003.docx]

S3 Table. Summary of *Triatoma dimidiata* population structure post-intervention (May-October 2022) by arm.Males=**♂**, Females**= ♀**.

| **Study arms** | **Adults collected** | | **Total**  **adults (%)** | **Nymphs collected** | | | | | **Total**  **Nymphs (%)** | **Total by arm (%)** |
| --- | --- | --- | --- | --- | --- | --- | --- | --- | --- | --- |
|  | **♂ (%)** | **♀ (%)** |  | **NI** | **NII** | **NIII** | **NIV** | **NV** |  |  |
| **Control** | 14 | 7 | 21 (100) | 1 | 7 | 16 | 30 | 44 | 98 (94.3) | 119 (92.2) |
| **Treatment** | 2 | 2 | 4 (100) | 1 | 1 | 2 | 0 | 2 | 6 (5.7) | 10 (7.8) |
| **Total** | **16 (64)** | **9 (36)** | **25 (100)** | **2** | **8** | **18** | **30** | **46** | **104 (100)** | **129 (100)** |
